# Dr. Addison W. Scott

**Published:** 1908-12

**Authors:** 


Dr. Addison W. Scott, of Syracuse, N. Y., died at the home of his daughter at Germantown, Philadelphia, October 24, 1908, from cancer of the stomach and liver, aged 57 years. He graduated at the Medical Department, University of Buffalo, 1890.

